# Epothilone B Facilitates Peripheral Nerve Regeneration by Promoting Autophagy and Migration in Schwann Cells

**DOI:** 10.3389/fncel.2020.00143

**Published:** 2020-05-26

**Authors:** Jianhua Zhou, Shengyou Li, Jianbo Gao, Yawei Hu, Shaochu Chen, Xinle Luo, Hao Zhang, Zhuojing Luo, Jinghui Huang

**Affiliations:** ^1^Department of Spine Surgery, The People’s Hospital of Longhua District of Shenzhen, Shenzhen, China; ^2^Department of Orthopedics, Xijing Hospital, Fourth Military Medical University, Xi’an, China

**Keywords:** epothilone B, autophagy, migration, peripheral nerve injury, remyelination

## Abstract

The search for drugs that can facilitate axonal regeneration and elongation following peripheral nerve injury has been an area of increasing interest in recent years. Epothilone B (EpoB) is an FDA-approved antineoplastic agent, which shows the capacity to induce α-tubulin polymerization and to improve the stability of microtubules. Recently, it has been increasingly recognized that EpoB has a regenerative effect in the central nervous system. However, the information currently available regarding the potential therapeutic effect of EpoB on peripheral nerve regeneration is limited. Here, we used a rat sciatic crush injury model system to determine that EpoB strikingly improved axonal regeneration and recovery of function. Also, EpoB (1 nM) did not result in significant apoptosis in Schwann cells (SCs) and showed little effect on their viability either. Interestingly, EpoB (1 nM) significantly enhanced migration in SCs, which was inhibited by autophagy inhibitors 3-methyladenine (3-MA). Since PI3K/Akt signaling has been implicated in regulating autophagy, we further examined the involvement of PI3K/Akt in the process of EpoB-induced SC migration. We found that EpoB (1 nM) significantly inhibited phosphorylation of PI3K and Akt in SCs. Further studies showed that both EpoB-enhanced migration and autophagy were increased/inhibited by inhibition/activation of PI3K/Akt signaling with LY294002 or IGF-1. In conclusion, EpoB can promote axonal regeneration following peripheral nerve injury by enhancing the migration of SCs, with this activity being controlled by PI3K/Akt signaling-mediated autophagy in SCs. This underscores the potential therapeutic value of EpoB in enhancing regeneration and functional recovery in cases of peripheral nerve injury.

## Introduction

Peripheral nerve injuries (PNIs) occur relatively frequently as a result of inflammatory, infectious, traumatic, or iatrogenic causes, resulting in chronic morbidity. PNIs are thought to affect millions of individuals globally each year, resulting in substantial social and economic burdens and rates of disability (Yu et al., 2015). PNI can lead to sensory or motor dysfunction as well as to tissue atrophy that cannot be reversed (Chen et al., 2019). While there are biological mechanisms capable of facilitating spontaneous recovery within the adult mammalian peripheral nervous system, the process is complex, and often results in unsatisfactory outcomes (Chen et al., 2015). The current gold standard treatments for PNIs are thus combinations of surgical repair, nerve bridging, and nerve grafting (Meyer et al., 2016). However, surgical procedures only restore the pathway through which regenerated axons reach their target organs (Huang et al., 2012; Chen et al., 2015) and do not improve the regenerative capability of the injured neurons. It is thus vital to explore drugs capable of promoting nerve regeneration to allow restoration of function after PNI.

Epothilone B (EpoB) is an antineoplastic agent with FDA approval, which shows the capacity to improve the stability of microtubules and to promote α-tubulin polymerization (Ballatore et al., 2012). EpoB can promote OV-90 and ABT-737 ovarian cancer cells apoptotic death *via* respective signaling through the Apo-2L/TRAIL and PI3K/Akt/mTOR pathways (Rogalska and Marczak, 2015; Li et al., 2016). More recently, EpoB has exhibited a regenerative effect in the central nervous system. Systemic administration of subtoxic EpoB doses enhances axonal regeneration and attenuates fibrotic scarring following an injury to the spinal cord by abrogating meningeal fibroblast polarization and migration (Ruschel et al., 2015). Intraperitoneal low-dose EpoB administration has been shown to suppress axonal microtubule deterioration and improve cognitive function in a murine tauopathy model (Ballatore et al., 2012). Furthermore, systemic EpoD administration has been suggested to protect against a murine MPTP-induced parkinsonism model (Cartelli et al., 2013). A recent study found that EpoB alleviates nigrostriatal pathway damage while enhancing motor functionality following intracerebral hemorrhage in mice (Yang et al., 2018). Taken together, these results confirm that EpoB has a regenerative effect in the central nervous system. However, reports of the effect of EpoB on nerve regeneration after nerve injury in the peripheral nervous system have been lacking.

Herein, we conducted a systematic assessment of how EpoB affects axonal regeneration and remyelination in a rat model of PNI. We explored the effect of EpoB on Schwann cells (SCs) and further revealed the underlying mechanisms. Our results show that the administration of EpoB improves axonal regeneration and functional recovery in a rat model of sciatic crush injury. We further show that EpoB (1 nM) significantly enhances migration in SCs, which is controlled *via* PI3K/Akt signaling-mediated autophagy. This work highlights the potential therapeutic value of EpoB in enhancing regeneration and functional recovery in PNI.

## Materials and Methods

### PNI Modeling

Adult male Sprague–Dawley (SD) rats (200–250 g) were obtained from the Laboratory Animal Center of the Fourth Military Medical University. Animals were housed following the NIH Guide for the Care and Use of Laboratory Animals, with the Animal Experimentation Ethics Committee of The Fourth Military Medical University having approved all studies described herein. Briefly, five rats were housed in each cage in a climate-controlled facility (23 ± 2°C; 35%–60% humidity; 12 h light/dark cycle).

The rat sciatic nerve injury model was generated as described in prior studies, with small modifications (Li R. et al., 2017). Briefly, 40 mg/kg of 1% pentobarbital sodium was i.p. injected into animals to achieve anesthesia, after which the left sciatic nerve was exposed through the generation of a 1 cm cut in the mid-thigh. Two vascular clips (30 g for 50 s; Oscar, Guangzhou, China) were used to compress the sciatic nerve, thereby inducing a moderate crushing injury. One of these clips was located 2 mm distal to the lesion, with the other being 7 mm proximal to the trifurcation of the sciatic nerve. The wound site was next closed using nondegradable sutures. Animals were then randomized into two model treatment groups: (1) control; and (2) EpoB (*n* = 10 per group). Sham controls underwent identical surgery but were not subjected to a crush injury. Animals in the EpoB group were administered 1 ml of 150 μg/ml EpoB *via* intraperitoneal injection for 7 days (once/day) following injury. Identical volumes of saline were administered to all other animals.

### Walking Track Assessment

At 1, 2, 3, and 4 weeks post-injury, the behavior of rats was evaluated *via* walking track assessment. Briefly, animals were allowed to walk down a wooden alley (5.0 × 8.0 × 45.0 cm^3^) that had a dark goal box positioned at the far end. To monitor walking behavior, the plantar surfaces of these rat’s feet were coated with acrylic paint, and white paper was used to cover the surface of the wooden alley. This enabled the visualization of anatomical landmarks within the resultant footprints. The prints were analyzed and utilized for the calculation of the sciatic functional index (SFI). This was repeated three more times to ensure footprints were clear enough for utilization.

### Von Frey Hair Test

Von Frey hair test was performed for all animals at 1, 2, 3, and 4 weeks post-surgery. Before testing animals were given 1 h to habituate to a glass cubicle, with the bottom of this container containing wire mesh and a surface filled with 5 × 5 mm^2^ holes that were 1 mm apart from one another. Rat tactile thresholds were evaluated by placing Von Frey filaments (NC12775; North Coast Medical, San Francisco, CA, USA) perpendicular to the planta pedis and pressing until a perceptible ~90° bend was evident for 6–8 s. At the threshold, the animal is expected to respond by quickly flicking its paw away from the hair. Forces sufficient to induce dodging reactions, such as the withdrawal of the paw, licking or avoidance behaviors, jittering, or the like were recorded as a means of calculating withdrawal threshold values. This was repeated thrice by the same investigator, with an interval of 15 min.

### Histological Analysis

**Figure 1:**
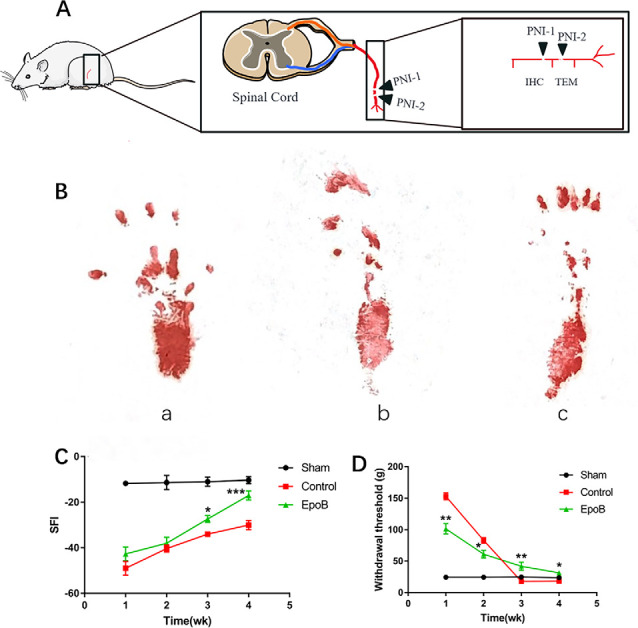
EpoB promotes locomotor and sensory recovery after nerve injury. **(A)** The schematic of Peripheral nerve injurie (PNI) model in the rats. **(B)** Representative images of walking track prints in the sham (a), control (b), and EpoB group (c) 4 weeks postoperatively. **(C)** The SFI values of each group at 1, 2, 3, and 4 weeks after surgery. **(D)** Withdrawal threshold assesse *via* the von Frey hair test at the predefined time points. *P < 0.05, **P < 0.01, and ***P < 0.001 vs. the control group. Data are presented as the mean ± SEM, *n* = 10 per group. Abbreviations: EpoB, epothilone B; SFI, sciatic functional index.

Isolated sciatic nerve portions containing the injured site were separated into two sections at 28 days after sciatic crush injury. One section (~1 cm; Figure 1A) was fixed overnight using cold 4% paraformaldehyde in PBS, followed by paraffin embedding and cutting into longitudinal sections for immunofluorescence staining. The sections were incubated with the primary antibodies at 4°C overnight. Primary antibodies were specific for NF-200 and MBP (Abcam, Cambridge, UK). Next, secondary antibodies were added for 1 h at room temperature (Cwbio; 1:10,000). Six sections per nerve group were analyzed as a means of assessing axonal regeneration *via* confocal fluorescent microscopy (A1+, Nikon, Japan) and at least six images in each nerve section were analyzed by the ImageJ software.

Another 2 mm segment (Figure 1A) was submerged in 3% glutaraldehyde, after which it underwent additional fixation with a solution of 1% osmium tetroxide in 0.1 M sodium cacodylate buffer (pH 7.3). Segments were then dehydrated, epoxy resin-embedded, and the distal nerve was cut to prepare transverse ultrathin sections (50 nm). Uranyl acetate and lead citrate were then used to stain these sections, followed by TEM (TECNAI Spirit, FEI, USA) evaluation. A total of six sections per nerve group were assessed at random to evaluate axonal regeneration, with six random fields of view per sample being analyzed. We measured myelin thickness (MT) and axonal diameter (AD) using the ImageJ software. Fiber diameter (FD) was calculated according to the formula: FD = 2 × MT + AD. The G-ratio (AD/FD) was also determined as a structural indicator including axonal myelination.

### Cell Culture and Immunofluorescence

Primary SCs were collected from newborn SD rats provided by the Laboratory Animal Center of the Fourth Military Medical University, according to previously published procedures (Xia et al., 2016). Briefly, animals were euthanized within 3 days of birth using cold alcohol (75%) followed by sciatic nerve extraction. The epineurium of the sciatic nerves was stripped and the endoneurium was combed under a microscope (Olympus, Tokyo, Japan). Scalpels were then used to mince these nerves into small pieces, after which 0.25% trypsin and 0.03% collagenase (Sigma–Aldrich, St. Louis, MO, USA) were used to digest them for 30 min at 37°C. Isolated cells were then cultured in DMEM (Thermo Fisher Scientific, Waltham, MA, USA) supplemented with 15% FCS (Thermo Fisher Scientific) and penicillin/streptomycin. At 24 h post-plating, cells were then treated for 48 h using cytosine arabinoside (10^−5^ M) as a means of preventing the proliferation of fibroblasts. Cells were then transferred to DMEM containing 2 mM forskolin (Sigma–Aldrich), 20 μg/ml bovine pituitary extract (Biomedical Technologies, MA, USA), and 15% FCS.

Cells were put into 6-well plates with coverslips for 24 h, followed by 4% PFA fixation and 0.15% Triton-mediated permeabilization. Then they were washed with PBS thrice and incubated using primary antibodies (1:100) overnight at 4°C. These antibodies were specific for S-100 and p75 (Abcam, Cambridge, UK). Next, secondary antibodies (Cwbio; 1:10,000) were added for 1 h, followed by additional washing using PBS and 4,6-diamidino-2-phenylindole (DAPI; Sigma–Aldrich) staining of cell nuclei. The ImageJ software was then used for enumerating p75NTR+ and S-100+ cells, revealing cell preparations to be highly pure (>96% SCs) as determined based upon the assessment of 5 random fields of view *via* confocal microscope (A1+, Nikon, Japan).

### Cell Viability Assay

SCs were added to 96-well plates (3,000/well, in quadruplicate). After 24 h, EpoB was added at appropriate concentrations for 24 h, after which each well was treated using 10-μl of Cell Counting Kit 8 (CCK8; Dojindo, Kumamoto, Japan) for 1 h at 37°C. Absorbance at 450 nm was then evaluated *via* a microplate reader.

### Apoptosis Assay

SCs were plated in 6-well plates (8 × 10^4^/well) for 24 h, after which fresh media with or without 1 nM EpoB was added for an additional 24 h. In some experiments, 3-methyladenine (3-MA; 0.5 mM), IGF-1 (100 ng/ml), or LY294002 (20 μM; MedChemExpress, Shanghai, China) was added to the cultures and then maintained in the same media for the entire experiment. Cells were then suspended in 195 μl Annexin V-FITC binding buffer to which 5 μl Annexin V-FITC and 10 μl PI (Beyotime, Shanghai, China) was added. Following a 20 min staining period, flow cytometry (Beckman Instruments, Pasadena, CA, USA) was used to analyze samples in triplicate.

### Cell Cycle Assay

PI (Beyotime) was used to evaluate cell cycle progression. Briefly, isolated SCs were fixed with 70% ethanol for 2 h at 4°C, after which PI was added before flow cytometric analysis (Beckman Instruments, Pasadena, CA, USA). The samples were assessed in triplicate.

### Migration Analysis

Transwell chambers (Corning, New York, NY, USA) with an 8 μm pore size were used to evaluate the impact of EpoB on SC migration. Briefly, SCs were added to the upper chamber (6 × 10^4^/well), while 500 μl of complete media supplemented with appropriate drugs was placed in the lower chamber. Following a 10 h incubation, a cotton swab was used to remove cells from the upper chamber surface, while remaining cells were fixed in 4% PFA and stained for 15 min using crystal violet. A total of five random fields of view per chamber were then assessed *via* microscopy (Olympus, Tokyo, Japan).

### Western Blotting

RIPA buffer (Beyotime) containing phosphatase and protease inhibitors was used to lyse cells for 5 min. The resultant lysates were spun for 15 min at 12,000 g, and a BCA Assay Kit (Beyotime) was used to quantify protein levels. Samples were then boiled for 15 min and separated *via* SDS-PAGE before transfer onto PVDF membranes (Thermo Fisher Scientific). Blots were then blocked using 5% non-fat milk in TBST, probed overnight with anti-p-PI3K (Cell Signaling Technology, Boston, MA, USA), anti-LC3B, anti-p62, anti-PI3K, anti-Akt, anti-p-Akt (Abcam, Cambridge, UK) and anti-β-actin (Cwbio, China) at 4°C, washed thrice in TBST, probed with secondary HRP-linked antibodies for 1 h, and assessed *via* chemiluminescent analysis (Bio-Rad). Samples were analyzed in triplicate.

### Transmission Electron Microscopy

A TEM (TECNAI Spirit, FEI, USA) was used to determine the numbers of autophagic vesicles in SCs. In brief, cells were put in fixative for transmission electron microscopy at 4°C for 2 h, washed thrice in PBS, and post-fixed for 2 h using 1% OsO_4_ in 0.1 M PBS (pH 7.4). Then OsO_4_ was removed from the sample, and it was dehydrated with an ethanol gradient with epoxy resin embedding. We conducted polymerization for 48 h at 60°C, followed by cutting into 70 nm ultrathin sections. Post-staining was next conducted for 15 min using uranyl acetate in pure ethanol, after which lead citrate was used and TEM (TECNAI Spirit, FEI, USA) was used for analysis. Data were quantified and analyzed by ImageJ software (50 cells were counted in a blind manner). At least three individual experiments were performed.

### Statistical Analysis

Data are means ± standard deviations (SDs) and were compared *via* one-way ANOVAs using SPSS 13.0 (IBM, IL, USA). Tukey’s *post hoc* test and Dunnett’s test were used to compare significant results (GraphPad Prism 7.0; GraphPad Software, CA, USA). *P* < 0.05 was the significance threshold.

## Results

### EpoB Promotes Functional Recovery Following PNI

To evaluate the impact of EpoB on peripheral nerve regeneration, rats with sciatic crush injury were intraperitoneally injected with 150 μg EpoB daily for 1 week. Locomotor recovery throughout the 4 weeks was evaluated by walking track analysis, which showed that the SFI progressively improved over time (Figure 1B). SFI did not differ significantly between the control and EpoB groups within the first 2 weeks. At 3 (−27.50 ± 1.58) and 4 (−17.32 ± 2.17) weeks after injury and treatment, the SFI in the EpoB group was significantly elevated relative to the control group (*P* < 0.05, −33.94 ± 1.06; 3 weeks post-injury; *P* < 0.05, −29.73 ± 1.97; 4 weeks post-injury), respectively (Figure 1C), suggesting superior motor functional recovery was achieved by the EpoB treatment.

Recovery of the sensory function of the injured hindlimb was evaluated throughout the 4 weeks using the Von Frey hair test. The results revealed that the withdrawal threshold in the EpoB group gradually decreased over 4 weeks, indicating that the recovery of sensory function was achieved by EpoB treatment after nerve crush injury. Also, the EpoB-treated rats showed a lower withdrawal threshold than the control rats within the first 2 weeks after injury (Figure 1D). The withdrawal threshold of the control group At 3 (17.61 ± 0.88) and 4 (17.92 ± 1.49) weeks after injury dramatically decreased to an even lower level than that of the sham-injury group (*P* < 0.05, 17.61 ± 0.88; 3 weeks after injury; *P* < 0.05, 17.92 ± 1.49; 4 weeks after injury), respectively (Figure 1D), indicating that hyperalgesia might be present after crush injury in the control group. Further investigations showed that the withdrawal threshold in the EpoB-treated group was significantly higher (*P* < 0.05, 43.21 ± 3.42; 3 weeks after injury; *P* < 0.05, 31.58 ± 2.81; 4 weeks after injury) than that in the control group at 3 (17.78 ± 1.64) and 4 (18.01 ± 1.33) weeks after injury (Figure 1D), indicating that EpoB is capable of ameliorating hyperalgesia at the later stage of nerve recovery. These findings suggest that EpoB treatment is capable of improving the recovery of sensory function after nerve crush injury.

### EpoB Enhances Axon Regeneration and Remyelination After PNI

**Figure 2:**
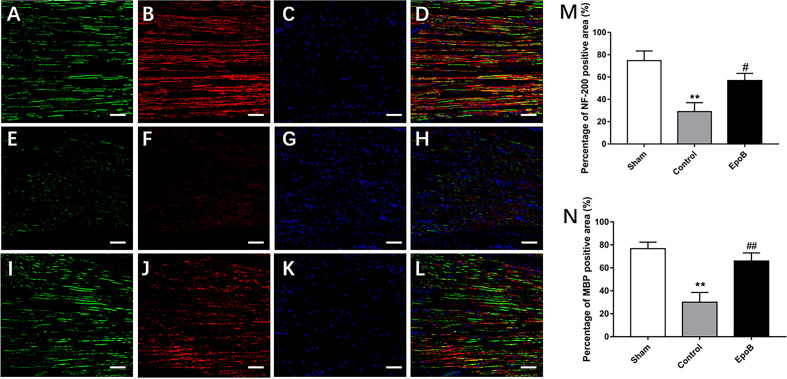
EpoB promotes regeneration of myelinated nerve fibers. Representative regenerated axon images in the nerves stained for NF-200 (green), MBP (red), and DAPI (blue) in the sham **(A–D)**, control **(E–H)** and EpoB **(I–L)** groups at 4 weeks after injury. Treatment of EpoB increased the ratio of positive area of NF-200 **(M)** and MBP **(N)**. Scale bars: **(A–L)** 20 μm. **P < 0.01 vs. the sham group; ^#^*P* < 0.05, ^##^*P* < 0.01 vs. the control group. Abbreviations: EpoB, epothilone B; MBP, myelin basic protein; DAPI, 4,6-diamidino-2-phenylindole.

We next examined the impact of EpoB on nerve regeneration, which is the structural foundation for functional recovery (Frendo et al., 2019; Liu et al., 2019). To evaluate the impact of EpoB on neuronal regeneration, we co-stained for NF-200 (a neurofilament heavy subunit found in both large and small axons) and myelin basic protein (MBP; which is a marker of myelination) was performed on longitudinal sections (Figures 2A–L). The ratio of the positive area of NF-200 (57.50% ± 3.35) and total positive of MBP (66.34% ± 2.74) in EpoB treated group was in the low range to that in the sham-treated group (73.81% ± 5.71; NF-200 and 77.39% ± 4.61; MBP), which was significantly higher than that in the PNI control group (29.51% ± 3.31; NF-200 and 29.82% ± 1.58; MBP; Figures 2M,N). These data indicated that the number of myelinated nerve fibers was significantly increased by EpoB compared to that in the PNI control group. All of these results suggest that EpoB administration contributes to the regeneration of myelinated nerve fibers after nerve crush injury.

**Figure 3:**
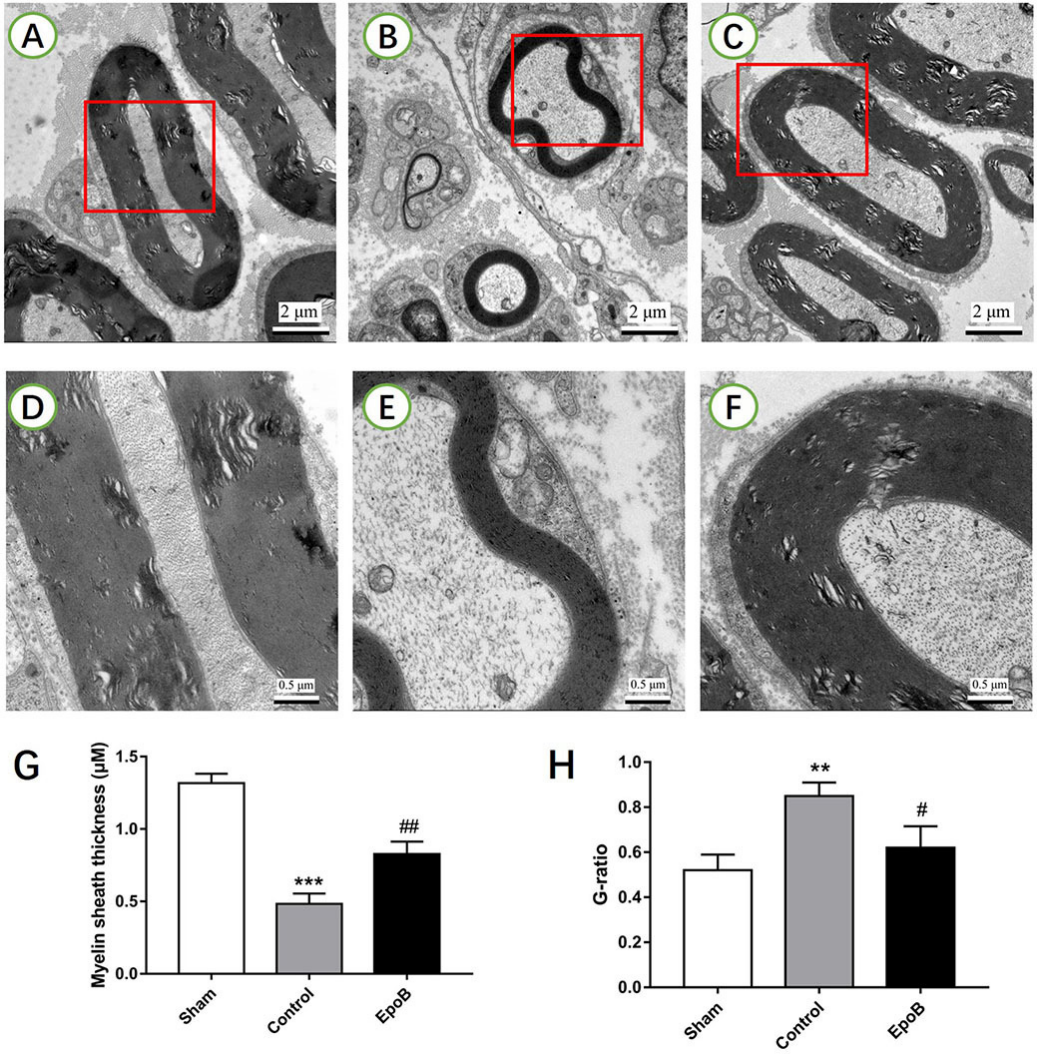
EpoB promotes sciatic nerve remyelination after nerve injury. Representative TEM images of regenerated axons **(A–C)** and myelin sheaths **(D–F)** in the nerve segment of the sham **(A,D)**, control **(B,E)** and EpoB **(C,F)** groups at 4 weeks after surgery, respectively. Quantification of the average myelin sheath thickness **(G)** and the G-ratio **(H)**. Scale bars: **(A–C)** 2 mm; **(D–F)** 0.5 mm. **P < 0.01 and ***P < 0.001 vs. the sham group; ^#^*P* < 0.05 and ^##^*P* < 0.01 vs. the control group. Abbreviations: EpoB, epothilone B; TEM, transmission electron microscopy; G-ratio, axon/fiber ratio.

The effect of EpoB on the myelination of regenerated axons was further evaluated by TEM (Figures 3A–F). A decreased thickness of the myelin sheath was observed in the PNI control group (*P* < 0.05, Figure 3G). In contrast, the thickness of the myelin sheath was significantly increased by EpoB treatment (*P* < 0.05; Figure 3G). Further morphometric analysis showed that EpoB treatment led to a significantly greater MT and a lower G-ratio (axon/fiber ratio) after nerve crush injury (*P* < 0.05; Figures 3G,H), indicated that EpoB is capable of improving remyelination of regenerated axons.

### The Effect of EpoB on the Biological Behaviors of SCs

**Figure 4:**
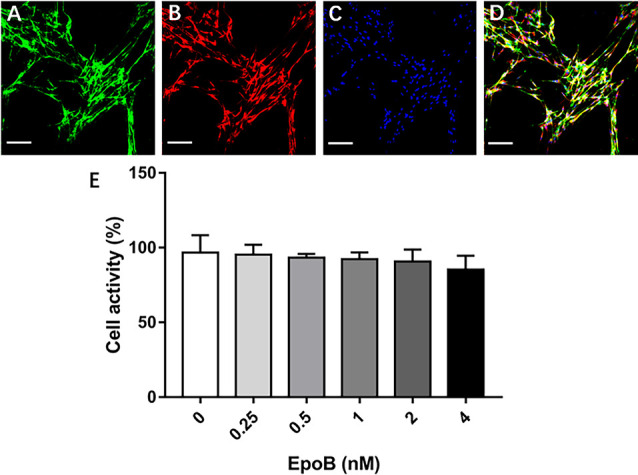
Identification of SCs and measurement of their viability. Double-immunofluorescent staining of cultured SCs showing expression of p75^NTR^ (green; **A**) and S-100 (red; **B**) with DAPI nuclear counterstaining (blue; **C**). **(D)** Merge images showed a SC purity >96%. **(E)** Effects of EpoB on SC viability assessed by the CCK-8 assay. Scale bar: **(A–D)** 100 μm. Abbreviations: EpoB, epothilone B; CCK-8, Cell Counting Kit 8; SC, Schwann cell; DAPI, 4,6-diamidino-2-phenylindole.

**Figure 5:**
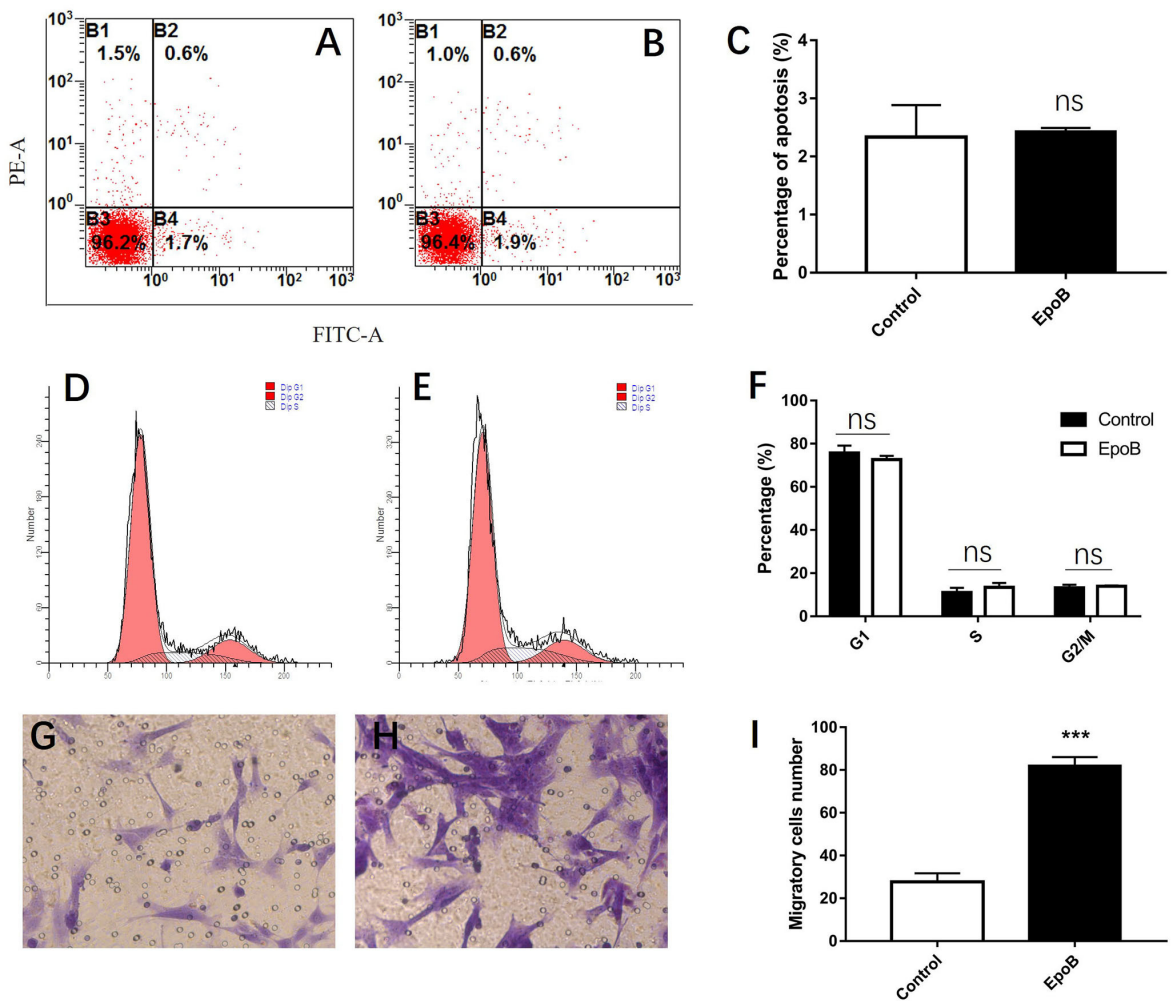
Effects of EpoB on SCs. EpoB has no effect on SC apoptosis **(A–C)** or the SC cell cycle **(D–F)** assessed by flow cytometry. **(G–I)** EpoB significantly promotes migration of SCs by in the transwell assay. ***P < 0.001 compared to the control group. Abbreviations: EpoB, epothilone B; SC, Schwann cell; ns, no significant.

We additionally explored the potential impact of EpoB on SCs in isolated SC cultures, which showed a purity of >96% (Figures 4A–D). EpoB (0.25–4.0 nM) had no significant effect on SC cell viability after 24 h of treatment (*P* > 0.05; Figure 4E). Therefore, EpoB at a moderate concentration (1 nM) was chosen for subsequent experiments. Further investigations showed that treatment with 1 nM EpoB did not result in significant SC apoptosis (Figures 5A–C), and showed little effect on the SC cell cycle (Figures 5D–F), which were performed by flow cytometry assay. These findings show that EpoB has no significant effect on the apoptosis and cycle of SCs. To our surprise, we found that EpoB (1 nM) dramatically enhanced the number of migrated SCs to 82 ± 4 when the Control grope increase to 28 ± 3 in the transwell migration assay (Figures 5G–I). These results indicate that EpoB is capable of promoting the migration of SCs.

### EpoB Promotes SCs Migration *via* PI3K/Akt Signaling-Mediated Autophagy

**Figure 6:**
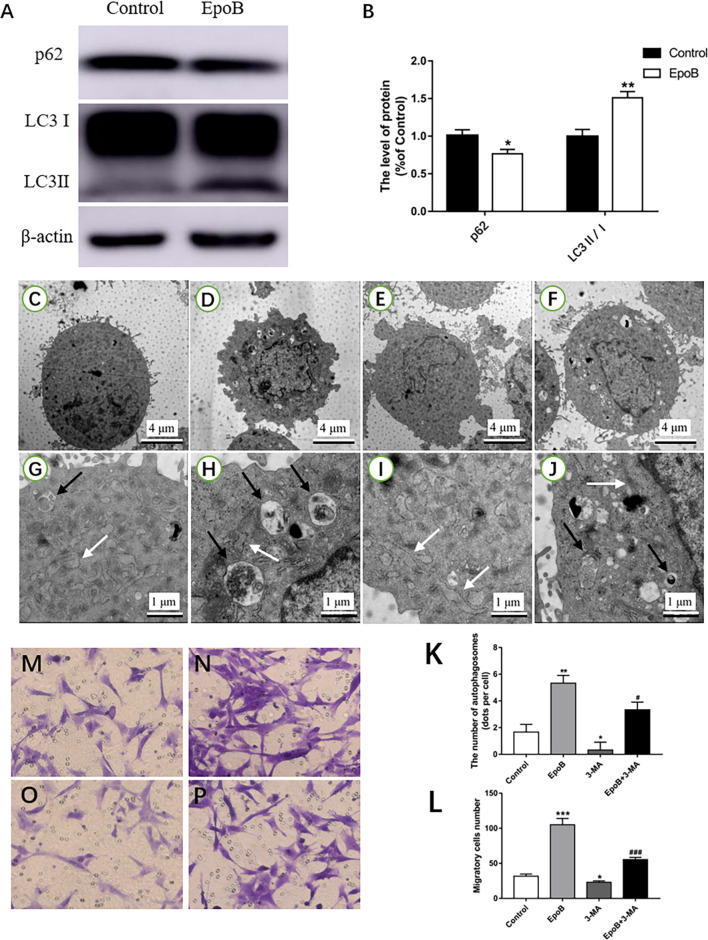
EpoB increases migration of SCs by promoting autophagy. Representative western blotting **(A)** and data analysis of LC3 and p62 in each group **(B)**. TEM analysis showing the presence of autophagosomes in SCs from the control **(C,G)**, EpoB **(D,H)**, 3-MA **(E,I)**, and EpoB+3-MA **(F,J)** groups. White arrowheads indicate normal endoplasmic reticula; black arrowheads indicate double-membraned autophagosomes. **(K)** Quantification of the total number of autophagosomes in each group. Transwell analysis of migratory cells in the control **(M)**, EpoB **(N)**, 3-MA **(O)**, and EpoB+3-MA **(P)** groups at 24 h after treatment. **(L)** Quantification of the total number of migratory cells in each group. Scale bars: **(C–F)** 4 μm; **(G–J)** 1 μm. *P < 0.05, **P < 0.01, and ***P < 0.001 compared to the control group; ^#^*P* < 0.05 and ^###^*P* < 0.001 compared to the EpoB group. Abbreviations: EpoB, epothilone B; 3-MA, 3-methyladenine; SCs, Schwann cells; TEM, transmission electron microscopy.

Autophagy has been implicated in cell migration. Herein, we examined the potential role of autophagy in EpoB-enhanced migration of SCs. We first examined whether EpoB could induce autophagy in SCs by western blot (Figure 6A). The results showed that EpoB significantly increased the level of LC3B II and reduced the level of P62 (two markers of autophagy) at 24 h after treatment (*P* < 0.05; Figure 6B). Also, transmission electron microscopy showed that EpoB markedly increased the number of autophagosomes (5 ± 1) in SCs after 24 h of the treatment compared to the control group (*P* < 0.05, 2 ± 1; Figures 6C–K). To further explore the role of autophagy in EpoB-enhanced migration of SCs, the effect of 3-MA (an autophagy inhibitor) on cell migration was examined (Figures 6C–J). It was found that 3-MA significantly inhibited EpoB-induced autophagosome production (*P* < 0.05; Figure 6K), and abolished EpoB-enhanced migration in SCs (*P* < 0.05; Figures 6L–P). Overall, these results suggest that autophagy was induced by EpoB and that this autophagy was involved in EpoB-enhanced migration in SCs.

**Figure 7:**
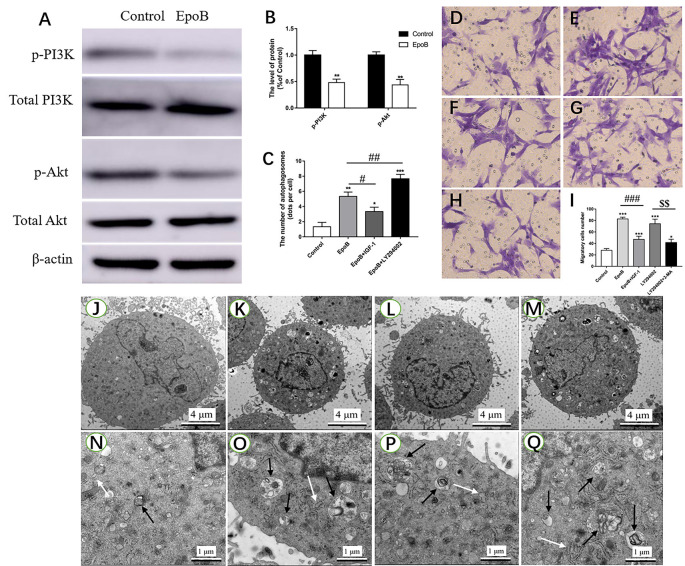
EpoB significantly enhances migration by PI3K/Akt signaling-mediated autophagy in SCs. Representative western blotting **(A)** and data analysis of p-PI3K and p-Akt in each group **(B)**. **(C)** Quantification of the total number of autophagosomes in each group. Transwell analysis of migratory cells in the control **(D)**, EpoB **(E)**, EpoB+IGF-1 **(F)**, LY294002 **(G)**, and LY294002+3-MA **(H)** groups at 24 h after treatment. **(I)** Quantification of the total number of migratory cells in each group. TEM analysis showing the formation of autophagosomes in SCs from the control **(J,N)**, EpoB **(K,O)**, EpoB+IGF-1 **(L,P)**, and EpoB+ LY294002 **(M,Q)** groups at 24 h after treatment. White arrowheads indicate normal endoplasmic reticula; black arrowheads indicate double-membraned autophagosomes. Scale bars: **(J–M)** 4 μm; **(N–Q)** 1 μm. *P < 0.05, **P < 0.01, and ***P < 0.001 compared to the control group; ^#^*P* < 0.05, ^##^*P* < 0.01, and ^###^*P* < 0.001 compared to the EpoB group, ^$$^*P* < 0.01 compared to the LY294002 group. Abbreviations: EpoB, epothilone B; 3-MA, 3-methyladenine; SCs, Schwann cells; TEM, transmission electron microscopy.

The PI3K/Akt signaling pathway has been shown to regulate autophagy in previous studies (Zhao et al., 2017; Ho and Gorski, 2019). In the present study, we further explored the role of PI3K/Akt signaling in autophagy-mediated migration by EpoB in SCs. First, it was found that EpoB significantly reduced the p-PI3K and p-Akt expression in SCs (*P* < 0.05; Figures 7A,B), suggesting that EpoB is capable of inhibiting the PI3K/Akt pathway. To further confirm the effect of PI3K/Akt signaling during EpoB-induced autophagy and migration in SCs, we evaluated the autophagy (Figures 7J–Q) and migration of SCs (Figures 7D–H) after treatment with a PI3K/Akt signaling enhancer (IGF-1; Wang L. et al., 2019) or inhibitor (LY294002; Li et al., 2019). It was found that IGF-1 significantly reduced the number of autophagosomes (*P* < 0.05; Figure 7C) and inhibited migration of SCs (*P* < 0.05; Figure 7I) induced by EpoB. In contrast, LY294002 further increased cell autophagy in the presence of EpoB (*P* < 0.05; Figure 7C). In addition, it was shown that LY294002 significantly increased migration in SCs; this effect was significantly reversed by the autophagy inhibitor 3-MA (*P* < 0.05; Figure 7I). Those findings show that PI3K/Akt signaling plays an important role in EpoB-induced autophagy, which is involved in EpoB-enhanced migration in SCs.

## Discussion

EpoB has been widely accepted as an antineoplastic agent (Shin and Kwon, 2017; Cheng et al., 2018). Recent studies have increasingly recognized its regenerative effect in the central nervous system. Herein, the role of EpoB in nerve regeneration, and the mechanism underlying this, were investigated in the peripheral nervous system for the first time. The conclusions of the present study can be summarized as follows: (1) EpoB promotes nerve regeneration and remyelination, and of enhances functional recovery after nerve crush injury, suggesting that EpoB has a strong regenerative effect in the peripheral nervous system; (2) EpoB strongly promotes SC migration, a process which involves EpoB-enhanced autophagy; (3) EpoB inhibits the PI3K/Akt signaling pathway, which regulates the autophagy-mediated migration of SCs by EpoB. All of these findings indicate that EpoB plays a regenerative role after nerve injury in the peripheral nervous system, highlighting its therapeutic value in enhancing regeneration and functional recovery in cases of PNI.

PNI is highly prevalent in clinical settings, and generally results in nonreversible tissue atrophy and dysfunction, thereby extolling a significant burden on affected individuals, their families, and their societies (Johnson et al., 2005). Although recent research has made great advances in approaches to peripheral nerve repair, including nerve transplantation and the construction of nerve transplants (Fujimaki et al., 2020), the prognosis of patients with PNI remains poor. Moreover, surgical procedures that restore the anatomical continuity of the injured peripheral nerve have advanced, but show little effect on improving the regenerative capability of the injured neurons. Therefore, the search for a novel therapy that can enhance axonal regeneration has become urgent, in order to improve functional recovery after PNI.

EpoB belongs to the epothilone class of drugs, which are the group of microtubule-stabilizing agents. At present, EpoB has been approved for cancer therapy in clinics (Brogdon et al., 2014). Interestingly, many studies have identified the regenerative effect of EpoB in the central nervous system (Ballatore et al., 2012; Ruschel et al., 2015). In an animal model of spinal cord injury, it was found that systemic subtoxic EpoB dosing improves axonal regeneration while also attenuating fibrotic scarring (Ruschel et al., 2015). Similarly, in a mouse model of intracerebral hemorrhage, EpoB has been shown to improve the integrity of the nigrostriatal pathway neural circuit, increase the number of dopaminergic neurons, and enhance the recovery of fine motor function (Ruschel et al., 2015). Despite these findings relating to the central nervous system, the effect of EpoB on peripheral nerve regeneration has not been studied thus far. In the present study, we found that EpoB is capable of promoting nerve regeneration and remyelination, and of enhancing function recovery after nerve crush injury. This suggests that EpoB has a strong regenerative effect in the peripheral nervous system. However, the mechanisms underlying the beneficial effect of EpoB on nerve regeneration in the peripheral nervous system are unclear.

It was reported that Wallerian degeneration occurs after peripheral nerve injury. Axonal lesions undergo degeneration and fragmentation, with myelin undergoing transformation into a neutral fat that can be taken up by macrophages. Axonal and myelin sheath debris also produce ovoids that are eventually broken down until no longer evident (Campbell, 2008). SCs are glial cells of the peripheral nervous system, which are capable of enhancing nerve regeneration in both the peripheral and central nervous system (Aszmann et al., 2008; Novikova et al., 2008). SCs play a key role in facilitating the regeneration of nervous system tissue given that they serve as a soruce of bioactive compounds that can support axonal migration and the release of compounds capable of controlling axonal outgrowth and remyelination (Zhang et al., 2009). In addition, the migratory ability of SCs has been widely accepted as a key process for guiding axonal elongation. In PNI, especially in peripheral nerve gap injury, axonal regeneration *in vivo* is limited by the extent of SC migration (Torigoe et al., 1996). Therefore, many studies have attempted to evaluate the feasibility of enhancing peripheral nerve regeneration by promoting SC migration. It has been shown that matrix metalloproteinase 7 (MMP-7) promotes axonal regeneration and remyelination by enhancing SC migration after sciatic nerve injury in rats (Wang H. et al., 2019). In addition, enhancing SC migration using concentration gradients of laminin-derived peptides has been shown to support axonal regeneration over a large nerve gap, further confirming the role of SC migration in axonal regeneration (Motta et al., 2019). Herein, to our surprise, EpoB (1 nM) showed little effect on the viability or apoptosis of SCs, but significantly enhanced SC migration. This indicates that EpoB might promote axonal regeneration by enhancing SC migration.

Autophagy is a key pathway that controls the breakdown of proteins and organelles, and it plays key roles in maintaining approrpaite cellular homeostasis *via* controlling catabolic activities in response to stress to improve energy and amino acid availability (Mizushima et al., 2008). Autophagy is associated with Alzheimer’s disease, Huntington’s disease and Parkinson’s disease (Bar-Yosef et al., 2019). In addition, autophagy is critical in the remodeling of presynaptic and postsynaptic constituents to sustain neuronal function (Gerónimo-Olvera and Massieu, 2019). An increasing number of studies have identified the role of autophagy in regulating cell migration, with diverse functions in different cell types (Zhao et al., 2017). Transforming growth factor-β1 (TGF-β1) promotes cellular invasion and migration by inducing autophagy in breast cancer cells (Huang et al., 2018), while *let-7* activates radial migration of newborn neurons *via* enhancing autophagy (Petri et al., 2017). In contrast, metformin has been found to enhance levels of autophagy and inhibit the migration of endothelial progenitor cells (Li W. D. et al., 2017). In a recent study, stromal cell-derived factor (SDF-1α or CXCL12) showed a pivotal role in regulating SC migration by enhancing autophagy after facial nerve injury (Gao et al., 2019). We found that EpoB induced autophagy in SCs, and that inhibition of autophagy by 3-MA was capable of inhibiting the EpoB-enhanced SC migration. All of these findings suggest that EpoB might promote SC migration by enhancing autophagy.

The molecules and pathways associated with regulating autophagy in SCs are still unclear. The PI3K/Akt/mTOR signaling pathway plays key roles in controlling cell proliferation, differentiation, growth, survival, and metabolism (Yu and Cui, 2016). PI3K/Akt signaling plays a critical role in maintaining intracellular stability by weakening the expression of neuronal apoptosis-related downstream molecules, such as cleaved caspase-3, Bcl-2 and Bax. This results in Bcl-2/Beclin-1 complex dissociation and subsequently leads to the autophagy response (Gao et al., 2016; Miao et al., 2016). It has been reported that the PI3K kinase inhibitor LY294002 is capable of blocking autophagosome formation (Du et al., 2016). In addition, sevoflurane post-conditioning has been found to attenuate traumatic brain injury-induced neuronal apoptosis by promoting autophagy *via* the PI3K/Akt signaling pathway (He et al., 2018). However, thus far, the role of the PI3K/Akt pathway in regulating autophagy in SCs has been unclear. In the present study, we examined the role of PI3K/Akt signaling in autophagy-mediated SC migration by EpoB. We found that EpoB treatment results in the inactivation of the PI3K/Akt signaling pathway. Further investigations showed that IGF-1, a PI3K/Akt pathway enhancer, is capable of inhibiting the autophagy and migration of SCs induced by EpoB. In contrast, LY294002 (a PI3K/Akt pathway inhibitor) was found to increase cell migration, an effect which was reversed by the autophagy inhibitor 3-MA. Circ-Spidr downregulation has been found to suppress DRG neuron axonal regeneration following injury to the sciatic nerve, partially *via* modulation of PI3K-Akt signaling (Mao et al., 2019). Maybe EpoB increase axonal regeneration of DRG neurons *via* the inhibiting of PI3K/Akt pathway immediately, which needs further research. Thus, these findings raise the posibility that PI3K/Akt signaling plays a key role in EpoB-induced autophagy, which is involved in EpoB-enhanced SC migration.

## Conclusion

In the present study, it was found that EpoB is capable of promoting axonal regeneration and remyelination, as well as functional recovery in a rat model of nerve crush injury. The beneficial effect of EpoB in the peripheral nervous system was attributable to enhanced SC migration, which was regulated by PI3K/Akt signaling-mediated autophagy in SCs. These results underscore the potential therapeutic value of EpoB in enhancing regeneration and functional recovery in cases of PNI.

## Data Availability Statement

All datasets generated for the present study are included in the article.

## Ethics Statement

The animal study was reviewed and approved by the Animal Experimentation Ethics Committee of The Fourth Military Medical University, People’s Republic of China.

## Author Contributions

JH, ZL, and HZ conceived and designed the experiments. JZ wrote the manuscript. SL and JG performed the experiments and analyzed the results. YH and SC isolated SCs from newborn Sprague–Dawley rats and analyzed the purity of the SCs. All authors revised and approved the final version of the manuscript.

## Conflict of Interest

The authors declare that the research was conducted in the absence of any commercial or financial relationships that could be construed as a potential conflict of interest.
