# Corrigendum: Epothilone B facilitates peripheral nerve regeneration by promoting autophagy and migration in Schwann cells

**DOI:** 10.3389/fncel.2025.1574709

**Published:** 2025-03-25

**Authors:** Jianhua Zhou, Shengyou Li, Jianbo Gao, Yawei Hu, Shaochu Chen, Xinle Luo, Hao Zhang, Zhuojing Luo, Jinghui Huang

**Affiliations:** ^1^Department of Spine Surgery, The People's Hospital of Longhua District of Shenzhen, Shenzhen, China; ^2^Department of Orthopedics, Xijing Hospital, Fourth Military Medical University, Xi'an, China

**Keywords:** epothilone B, autophagy, migration, peripheral nerve injury, remyelination

In the published article, there were errors in Figures 3, 5 as published. Figure 3D, intended to serve as an enlarged representation of Figure 3C, was placed in the wrong position. Although the trend of the result is accurate, the image does not correspond to the experiment. To rectify this, the authors have reorganized the transmission electron microscopy images in Figure 3. Additionally, the transwell images in Figures 5H, 6N appear to overlap. Although both images represent results from the same experimental group (SCs treated with EpoB), the authors have chosen to replace Figure 5H to avoid any possible confusion. The corrected Figures 3, 5 and their respective captions appear below.

**Figure 3:**
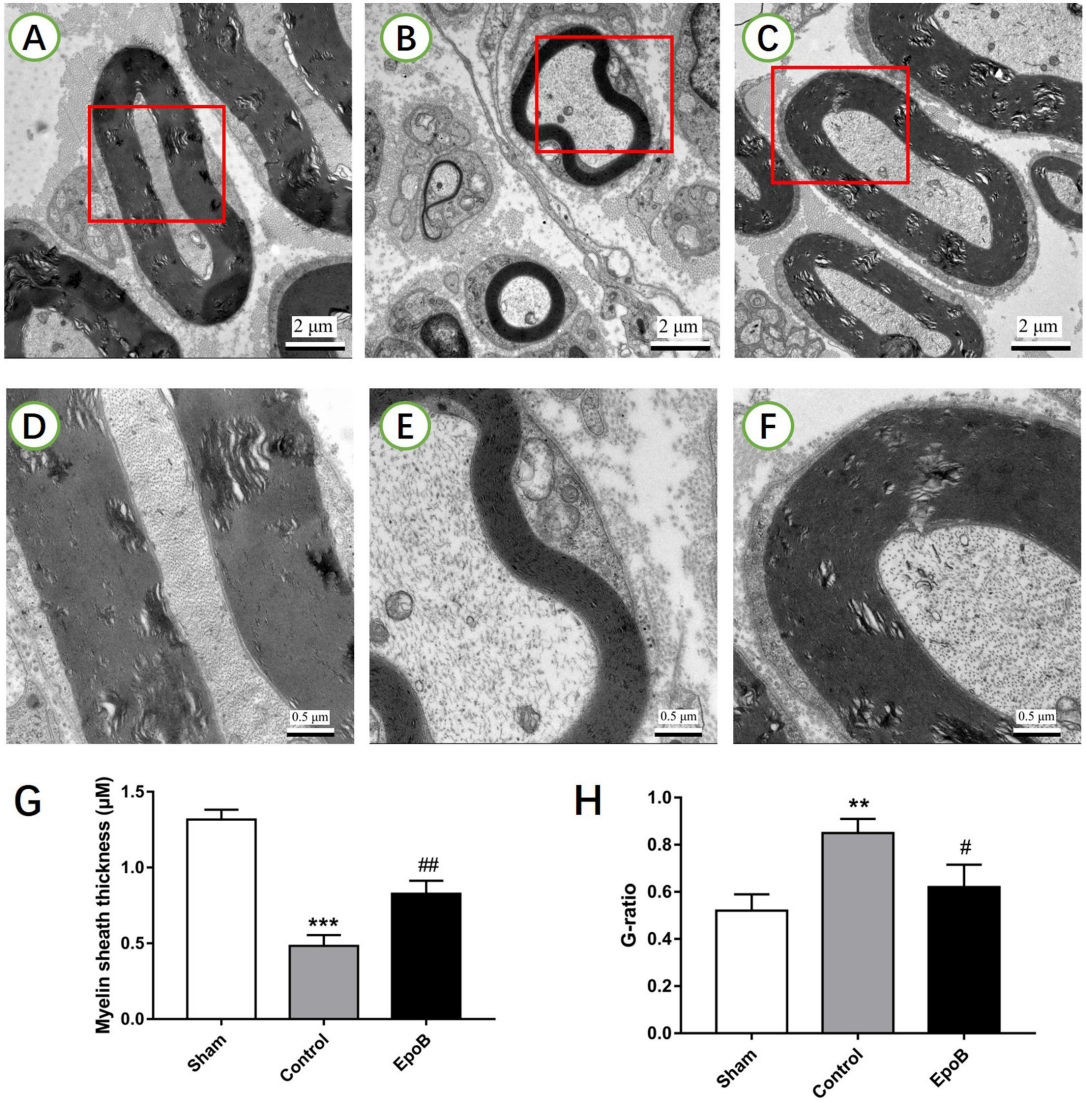
EpoB promotes sciatic nerve remyelination after nerve injury. Representative TEM images of regenerated axons **(A–C)** and myelin sheaths **(D–F)** in the nerve segment of the sham **(A, D)**, control **(B, E)**, and EpoB **(C, F)** groups at 4 weeks after surgery, respectively. Quantification of the average myelin sheath thickness **(G)** and the G-ratio **(H)**. Scale bars: **(A–C)** 2 mm; **(D–F)** 0.5 mm. ^**^P < 0.01 and ^***^P < 0.001 vs. the sham group; ^#^*P* < 0.05 and ^##^*P* < 0.01 vs. the control group. EpoB, epothilone B; TEM, transmission electron microscopy; G-ratio, axon/fiber ratio.

**Figure 5:**
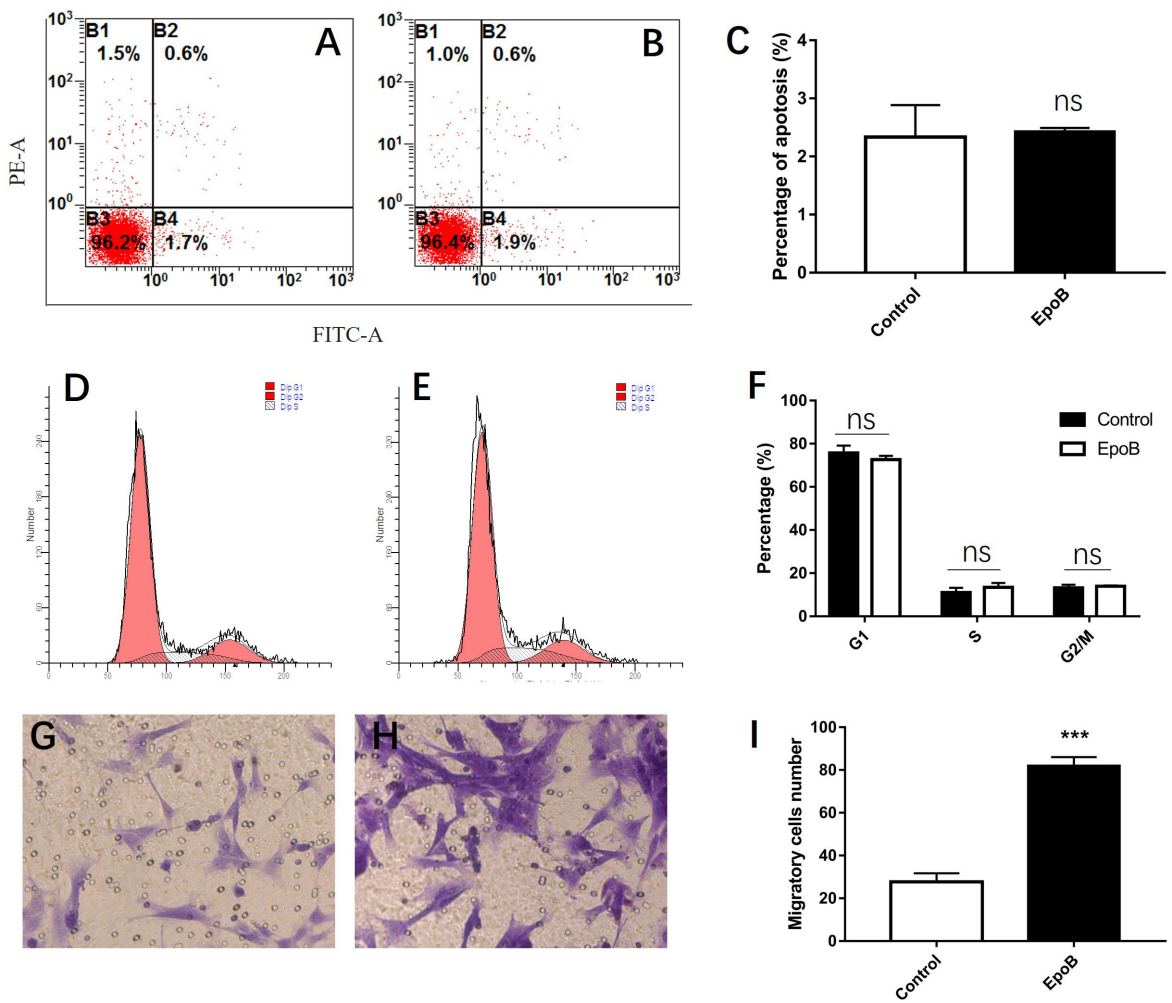
Effects of EpoB on SCs. EpoB has no effect on SC apoptosis **(A–C)** or the SC cell cycle **(D–F)** assessed by flow cytometry. **(G–I)** EpoB significantly promotes migration of SCs by in the transwell assay. ^***^P < 0.001 compared to the control group. EpoB, epothilone B; SC, Schwann cell; ns, no significant.

The authors apologize for these errors and state that they do not change the scientific conclusions of the article in any way. The original article has been updated.

